# Supplementary material for: Are carbon emissions trading and green financial instruments synergistic? -Comprehensive quantitative research based on content analysis
Source: PLoS One. 2024 Mar 7;19(3):e0298601. doi: 10.1371/journal.pone.0298601 (PMC10919601; doi:10.1371/journal.pone.0298601)
Supplement: S1 Data — (ZIP) [file pone.0298601.s001.zip › Data/地级市Z_F工作报告.zip!/║╙▒▒/╠╞╔╜╩╨/└┤╫╘╣1⁄2╓┌║┼í╛┬φ┐╦╩2╛▌═°í┐_2012.docx]

一、2011年工作回顾

刚刚过去的一年，是实施“十二五”规划的第一年。面对错综复杂的形势和各种严峻挑战，全市上下在市委的坚强领导下，全面落实中央、省一系列决策部署，坚持以科学发展为主题，以加快转变经济发展方式为主线，全力做好稳增长、调结构、控物价、惠民生、促和谐各项工作，较好地完成了市十三届人大四次会议确定的各项目标任务，实现了“十二五”良好开局。

——综合经济实力跨上新台阶。

全市地区生产总值达到5442.4亿元，同比增长11.7%。全部财政收入由上年的439亿元跃升到555.5亿元，其中一般预算收入255.6亿元，分别增长26.6%和30.5%，收入总量、增量继续稳居全省首位，全部财政收入占地区生产总值比重、一般预算收入占全部财政收入比重达到10.2%和46%，分别提高0.4个和1.4个百分点。全社会固定资产投资完成2546.1亿元，增长6.2%，470个项目开工建设，120个项目竣工投产或投入使用。规模以上工业增加值完成2835.8亿元，增长15.6%。社会消费品零售总额达到1316.2亿元，增长17.6%。

——产业结构得到新优化。

国家级信息化与工业化融合试验区顺利通过国家验收。实施传统产业技术改造项目450项，完成工业技改投资620亿元，占工业投资的59.8%;推进传统产业资本战略性转移项目105个，完成投资176亿元。渤海钢铁集团整合重组纳入国家钢铁工业“十二五”规划，钢铁前五强企业(集团)生产集中度达到68%，精品钢比重63%，分别提高2个和3个百分点。装备制造业增加值353亿元，占规模以上工业的12.5%，同比提高3.4个百分点。高新技术产业实现增加值126.7亿元、增长20%，高速动车组、矿用抢险探测机器人等战略性新兴产业正在加速产业化，纳米发电机等一批高科技项目引进落地。服务业完成投资1404.8亿元，占全社会固定资产投资的55.2%。年初确定的10大旅游和10大交通项目进展顺利。科技创新能力提高，市级以上科研机构发展到120家、比上年增加20家，新增高新技术企业17家，总数达到80家。工业聚集区建设步伐加快，年主营业务收入超百亿元园区达到8个。节能减排工作深入推进，新淘汰落后产能铁163万吨、钢1032万吨、水泥2215万吨、炼焦145万吨、造纸40.8万吨，预计万元生产总值能耗下降4.13%，化学需氧量、二氧化硫排放量分别削减1.5%，可完成省达目标任务。

——以曹妃甸为龙头的沿海开发取得新进展。

沿海地区完成生产总值1620亿元，增长13.5%，增速高于全市1.8个百分点。曹妃甸新区完成投资600.9亿元。曹妃甸矿石码头二期、通用码头三期和通用散货码头投入试运营，唐山港全年货物吞吐量达到3.1亿吨，增长20%，在全国港口排名由上年的第9位上升到第8位。华电曹妃甸重工装备制造基地、汽车零部件等一批产业项目完工或开工建设，首钢京唐钢铁二期和华润曹妃甸2×100万千瓦发电机组项目纳入国家“十二五”规划并全面启动前期工作，中石化千万吨炼油项目已由国家发改委委托中咨公司开展项目评估。曹妃甸国家级经济技术开发区和综合保税区申报取得阶段性成果。中日唐山曹妃甸生态工业园建设取得积极进展。工业区综合服务区二期、三期工程相继开工。唐山湾生态城12平方公里起步区路网初步形成，企业生活服务基地等基础配套设施建设取得新进展。

——新农村建设呈现新气象。

全面落实各项强农惠农政策，发放粮食直补、综合直补等惠农资金8.3亿元。粮食生产再获丰收，粮食总产达317.8万吨，增长2.5%，实现“八连增”。全年投入农田水利资金3.8亿元，新增灌溉面积1.6万亩，新建节水灌溉面积34万亩。实施龙头企业壮大、品牌农业创新、粮食核心区建设等16项重点工程，新培育国家级农业龙头企业2家、省级16家，农民专业合作社达到1123个，农业产业化经营率65%，比上年提高1个百分点。农村劳动力向非农产业转移15万人，非农收入占农民人均纯收入比重达到73%。新开工省级新民居建设示范村303个，累计达到735个，居全省首位，农村面貌和人居环境有了新提升。

——城镇面貌发生新变化。

城镇建设三年上水平工作扎实开展，全年城镇建设完成投资超过1000亿元。作为城镇建设三年上水平“一号工程”的环城水系公园广场建设及周边区域改造进展顺利，36个公园广场已完工17个、在建10个，周边旧小区和城中村改造同步启动;南湖生态城西北片区回迁安置小区、影视基地、老唐山风情小镇等项目加快推进;凤凰新城新青少年宫、传媒大厦等公益项目及一批住宅、商业、总部基地项目加快建设;空港城完成总体规划编制，市政基础设施建设陆续启动。旧城改造步伐加快，万达广场、新华贸等一批重点区域改造项目建成。新改扩建南新道等9条城市道路，完成天然气管网完善、丰润热电市区联网续建、市区污泥处置厂等一批市政基础设施项目。205国道改造丰南段、京沈高速迁西支线主体工程通车。以创建全国文明城和国家生态园林城为契机，深入开展城市“绿、美、亮、净”综合整治，城市面貌进一步提升。县城扩容和小城镇建设加速。全市城镇化率达到52.5%，同比提高1.6个百分点。

——改革开放迈出新步伐。

医药卫生体制改革取得阶段性成果，基层医药卫生体制综合改革全面推开，基层医疗机构基本药物零差率销售全面实行。企业改革不断深化，开滦集团等5家企业入围全国500强，庞大集团成功上市，上市公司累计达到10家。事业单位改革稳步实施，乡镇机构改革全面完成。区域金融体系不断完善，新增域外金融机构8家、小额贷款公司11家、村镇银行2家。文化、科技、教育、社会管理等领域改革继续深化。民营经济发展活力进一步增强，增加值占全市生产总值比重达到66.9%，税金占全部财政收入比重达到69.1%。对外开放水平实现新提升。成功举办第二届曹妃甸临港产业国际投资贸易洽谈会、第十四届唐山中国陶瓷博览会等大型经贸交流活动。全年实际利用外资10.97亿美元，增长24%，法国赛诺菲、美国沃尔玛、香港新世界等一批战略投资者入驻唐山;进出口总额达到108.6亿美元，其中出口38.9亿美元，增长33.2%;对外投资总额1.6亿美元，增长45%。对内交流合作取得重要成果，引进市外资金731.5亿元，增长1.25倍。

——改善民生取得新成效。

投资160亿元，为群众办的20件实事全部完成。城镇居民人均可支配收入和农民人均纯收入达到21785元和9460元，分别增长11.4%和13.8%。就业形势总体稳定，城镇新增就业10.2万人，下岗失业人员实现再就业3.67万人，城镇登记失业率4%。城镇基本医疗、失业、工伤和养老保险覆盖范围进一步扩大;新农保制度实现全覆盖，参保人数达到312万人，72万农民按月领取养老金;新农合筹资标准由每人每年140元提高到230元，报销比例提高5个百分点，在全省率先启动新农合大病统筹基金。城乡低保标准由每人每月310元、每人每年1540元分别提高至363元和1900元，保障水平居全省首位。新开工震后危旧平房改造安置住房4.9万套，开工建设保障性安居工程9.89万套，全面完成省达任务，廉租住房保障总户数达到1.9万户。同时，坚持不懈狠抓安全生产和食品药品安全工作，保障了人民群众生命财产安全。2011年我市安全生产各项控制指标达到全国先进水平，被国务院安委办评为安全生产宣传教育工作优秀单位。带着感情做好群众工作，健全社会矛盾综合调控机制，社会治安综合治理不断强化，和谐稳定的社会局面进一步巩固。

——社会事业发展取得新进步。

学前三年教育毛入园率达到85.4%，义务教育均衡发展，在全省率先基本普及高中阶段教育，职业教育办学规模水平不断提升，河北联合大学通过博士学位授权点建设中期检查，唐山学院接受了教育部本科高校办学水平评估。公共卫生服务保障水平进一步提高，“健康唐山，幸福人民”行动深入开展，免费为全市70万45岁以上农民、城镇无业居民中的“三高”人群进行了体检，新工人医院和新妇幼医院开工建设，乡镇卫生院标准化率达到100%，村卫生室达标率85%。文化事业和文化产业加快发展，市博物馆改扩建工程竣工投入使用，市文化广场、新工人文化宫开工，“农家书屋”等文化惠民工程扎实推进，启新记忆文化创意产业园等一批文化产业项目顺利实施。全民健身运动广泛开展，在第七届全国城运会上我市获运动成绩和精神文明双丰收，市奥体中心开工建设。全面贯彻计划生育基本国策，人口自然增长率控制在4.3‰。第六届全国双拥模范城创建工作扎实推进。广播电视、外事侨务、民族宗教、慈善、残疾人、防震减灾、气象、人防、档案、地方志、国防动员、民兵预备役等各项事业取得新成绩。

——政府行政效能和服务水平实现新提升。

高度重视向人大常委会报告工作，自觉接受人大法律监督、政协民主监督和社会监督，办理人大代表建议和政协委员提案682件，按时办结率100%，代表、委员满意率99%。深入开展创先争优活动，大力推进政风建设。“五五”普法圆满完成，“六五”普法全面启动，被评为全国法制宣传教育先进城市。坚持依法行政，加强权力运行监控机制建设，扎实推进行政权力公开透明运行，深入开展行政机关晒权评议活动，惩治和预防腐败体系建设取得明显成效。

这里向各位代表再报告一下全国文明城市创建工作。早在1988年，我市就开始启动全国文明城市创建工作。经过历届市委、市政府和全市人民23年矢志不渝的拼搏努力，在去年12月份召开的全国精神文明建设工作表彰大会上，我市被中央文明委正式授予“第三批全国文明城市”称号。这对于进一步提升唐山的知名度和美誉度，增强全市人民的自豪感和荣誉感，在更高起点上推动城市发展,具有重要意义。在创建过程中，全市各级组织和广大干部群众顾全大局，通力协作，攻坚克难，充分展示了唐山人民的良好精神风貌。全国文明城市的创建成功，必将激励广大干部群众为建设更加繁荣、更加文明、更加和谐的美好家园而不懈奋斗!

各位代表，过去的一年，在形势复杂多变的情况下，我市经济社会发展取得了令人鼓舞的新成绩。这是党中央国务院、省委省政府和市委正确领导的结果，是全市人民同心同德、共同奋斗的结果。在此，我代表市政府，向全市工人、农民、知识分子、各级干部和广大建设者，向人大代表、政协委员，向各民主党派、工商联、无党派人士和人民团体，向驻唐部队、武警官兵、公安政法干警以及关心支持唐山发展的各界朋友，表示崇高的敬意和衷心的感谢!

在看到成绩的同时，我们也清醒地认识到，我市经济社会发展中还面临着一些矛盾和问题。主要是：经济结构性矛盾仍然比较突出，科技创新能力和经济发展的质量效益有待提高，节能减排和环境保护任务艰巨;资金供给紧张，金融生态环境有待改善，部分企业生产经营遇到一些困难;市场物价高位运行，城乡居民生活受到影响;社会事业与经济发展还不相协调，保障和改善民生还有大量工作要做;政府行政效能和服务水平有待进一步提升，一些政府工作人员还不同程度地存在服务意识不强、效率不高、形式主义、官僚主义和消极腐败等现象。对这些问题，我们必须高度重视，采取有效措施，认真加以解决。

二、2012年工作思路和目标

2012年是深入实施“十二五”规划承上启下的重要一年，是我们全面贯彻落实省市党代会精神和省委对唐山提出“六个走在全省前面”新要求的起步之年，也是本届政府任期的最后一年。我们还将迎来党的十八大召开。做好今年的政府工作，意义重大。

展望2012年，我们既面临着诸多严峻挑战，同时更面临着许多机遇和有利条件。从挑战看，外部环境极其复杂，世界经济增速放缓，经济复苏的不稳定不确定因素增多;国内经济发展中不平衡、不协调、不稳定的问题还很多，经济增长下行与物价上涨压力并存;国家实施房地产调控政策，将对我市房地产、建材、钢铁等行业产生直接或间接影响;同时省内外兄弟城市都在竞相发展，使我市面临着加速转型和提速跨越的双重压力。从有利条件看，全球背景下的新科技革命和产业转移重组加速，我国经济发展的内在优势和长期向好的趋势没有改变，中央确定把“稳中求进”作为今年经济工作的主基调，重视扶持发展实体经济，有利于我们转方式调结构、实现经济平稳可持续发展。从唐山自身讲，做好今年工作有五个方面的有利条件：一是迎来了重大机遇。河北沿海地区发展规划上升为国家战略，京津冀区域经济一体化、首都经济圈纳入国家“十二五”规划，为我市聚集优质生产要素、促进现代产业发展创造了有利条件。二是拥有强大的动力。省委、省政府对唐山的发展高度重视，明确提出“六个走在全省前面”的新要求，为我们在新的起点上谋求新的发展注入了强大动力。三是打造了强劲支撑。我市不仅拥有沿海、港口、资源、产业等独特优势，形成了较为雄厚的经济基础，而且培育了一批新的经济增长点和成长性好的大企业，这将有力地拉动经济强劲增长。四是实现了良好开局。我市“十二五”开局之年保持又好又快发展势头，转方式、调结构取得积极成效，主要指标特别是质量效益指标明显提升，为做好今年乃至整个“十二五”时期工作奠定了坚实基础。五是形成了大好局面。全市上下加快发展的意识更加强烈、氛围更加浓厚、士气空前高涨，形成了推动更好更快更大发展的强大合力。对此，我们一定要统一思想认识，科学把握发展大势，坚定发展信心，抢抓发展机遇，积极应对挑战，稳中求进、以进促稳，奋发向上、大有作为，牢牢把握工作的主动权。

坚持以邓小平理论和“三个代表”重要思想为指导，深入贯彻落实科学发展观，全面落实中央、省和市委一系列工作部署，以科学发展为主题，以加快转变经济发展方式为主线，以调整经济结构为突破口，着力扩大内需，着力加快沿海开发开放，着力加强自主创新和节能减排，着力深化改革开放，着力保障和改善民生，着力加强和创新社会管理，努力实现更好更快更大发展，推动各项工作走在全省改革发展前面，以优异成绩迎接党的十八大胜利召开。

市第九次党代会科学描绘了唐山未来发展的宏伟蓝图，明确了今后五年的奋斗目标和主要任务，为推动唐山科学发展、争先进位指明了方向。去年年底召开的市委九届二次全会围绕落实中央、省部署和市第九次党代会精神，对2012年工作进行了全面部署。根据市第九次党代会和市委九届二次全会精神，今年政府工作的总体要求是：

全市经济社会发展的主要预期目标是：地区生产总值增长11%;全部财政收入增长15%，其中一般预算收入增长16%;全社会固定资产投资增长21%;城镇居民人均可支配收入和农民人均纯收入均增长10%以上;万元生产总值能耗降低率、主要污染物排放量降低率均完成省达目标要求;城镇登记失业率控制在4.5%以内;人口自然增长率控制在4.98‰以内;居民消费价格涨幅控制在4%左右。

上述目标，是从我市实际出发，综合考虑各种因素确定的，力求既体现转方式、调结构，推进科学发展的要求，又符合应对竞争、争先进位、努力走在全省改革发展前面的现实需要，既积极进取，又实事求是。

做好今年政府工作，我们要牢牢把握科学发展、争先进位这一总体目标，推动各项工作走在全省改革发展前面;牢牢把握稳中求进、以进促稳，奋发向上、大有作为这一工作主基调，努力实现更好更快更大发展;牢牢把握加快转变经济发展方式、调整经济结构这一中心任务，力促发展质量和速度双提升;牢牢把握曹妃甸开发建设这一战略重点，举全市之力加速打造沿海经济隆起带，培育新的增长极;牢牢把握深化改革开放这一关键举措，激活内力、广借外力，破瓶颈、求发展;牢牢把握城镇这一综合载体，推动城市建设上水平、出品位;牢牢把握保障和改善民生这一根本目的，真正让发展进步的成果惠及千家万户，让全市人民生活得更加幸福!

三、2012年主要工作任务

为实现今年经济社会发展各项目标任务，着力抓好十个方面的重点工作。

(一)着力推进重点项目建设，确保固定资产投资较快增长。

从唐山讲，现阶段保持经济增长仍然靠投资拉动。坚持把项目建设作为扩内需、稳增长的重要抓手，作为转方式、调结构、惠民生的重要载体，创新理念抓投资，凝心聚力上项目，努力增强投资对经济增长的拉动力。

优化投资结构。

坚持存量调强、增量调优，遵循国家产业政策，加大传统产业改造升级、战略性新兴产业、现代服务业、民生和社会事业、生态环保等领域投资力度，确保工业技改投资占工业投资的比重，装备制造业、高新技术产业和战略性新兴产业投资占工业投资的比重，服务业投资占全社会固定资产投资的比重，均有较大幅度提升。引导社会资本投资方向，鼓励支持传统产业资本向战略性新兴产业、现代服务业和产业链高端转移，年内传统产业资本转移力争突破200亿元。在抓好事关全局重大项目的同时，高度重视和加强中小项目建设，大中小项目一起抓、一二三产并举，形成大中小项目协调推进的良好局面。

狠抓重点项目建设。

全年安排重点项目200个、总投资4100亿元，年度计划投资1000亿元以上。其中，10亿元以上重大产业项目40个，年度计划投资150亿元。加快浙江物产物流基地等80个在建续建重点项目建设，确保如期投产达效;力促曹妃甸大型海水淡化、中煤科工煤机装备制造等120个重点项目开工。同时，加快推进晶源裕丰高端可通用芯片等120个前期项目，确保取得实质性进展。加强重点项目的谋划储备，全市重点项目入库数量保持在1000个以上。全面落实重点项目建设责任制，继续组织开展重点项目观摩活动，在全市上下营造比学赶超、抓项目促发展的浓厚氛围。

推动各类开发区和产业聚集区建设。

加快国家级高新技术产业开发区和10个省级开发区建设步伐，充分发挥其在对外开放、科技创新、产业聚集等方面的示范引领作用。进一步完善省级工业聚集区和各特色产业园区规划设计，加快园区路、水、电、气、讯等各项基础设施建设，为项目进区和产业聚集创造条件。制定工业聚集区发展扶持政策和管理办法，加速现代产业和优质生产要素向园区集中。今年，每个省级工业聚集区新开工重点项目10个以上，完成投资和主营业务收入分别增长25%和20%以上。

积极破解项目建设瓶颈制约。

优化国土资源管理，完善建设用地分配和使用管理办法，健全重大项目用地事前协商制度，积极争取增量、盘活存量土地，科学设置投资强度和产出效益，推进节约集约用地，努力保障项目用地需求。坚持依法依规用地，坚决严肃查处违法违规用地行为。积极争取金融信贷支持，全面整合财政资金，通过发行企业债券、激活民间资本、招商引资等多种途径，进一步拓宽融资渠道。

(二)着力推进产业结构优化升级，加快经济发展方式转变和经济结构调整。

坚持走新型工业化道路，按照“一产抓特色、二产抓提升、三产抓拓展”和“改造旧的、发展新的、培育好的”思路，加快推动产业结构优化升级，构建具有唐山特色的产业发展新格局。

深入推进传统产业改造提升。

全面推进工业提升计划，深入开展对标行动，组织实施100项传统产业技术改造重点项目，加快我市由钢铁大市向钢铁强市转变，由制造业大市向制造业强市转变，由工业大市向工业强市转变。围绕打造精品钢材基地，实施“钢铁行业优化提升工程”和“钢铁效益倍增计划”，严控钢铁产能总量，加快钢铁产业改造升级和整合重组，加大特种钢、专用钢等新产品开发力度，推进钢铁产业向精深加工和产业链高端发展，经过三至五年努力实现钢铁效益翻番。重点抓好渤海钢铁集团整合重组、津西钢板桩等项目，精品钢比重达到65%以上。围绕打造装备制造基地，重点支持高速动车、冶金机械、选煤机械等具有比较优势的企业和产品向集群化发展，加快发展技术含量高的高端装备制造等耗钢产业。围绕打造高端建材基地，支持冀东、惠达等优势骨干企业做大做强，鼓励新型建筑材料产业发展。围绕打造滨海化工基地，以曹妃甸工业区、海港开发区、南堡开发区为重点，推进煤化工、海洋化工、石油化工发展和产业链延伸。围绕打造绿色能源基地，加快推进煤炭、电力、石油、天然气等传统能源高效清洁开发利用。

加快发展战略性新兴产业。

以高端装备制造、新能源、新材料、节能环保、生物医药、新一代信息技术为重点，加强引导和政策扶持，积极培育开发科技主导型产业和产品，加快产业化步伐，努力形成新的产业优势。重点抓好中低速磁悬浮列车、纳米发电机、系列机器人、电动汽车、单(多)晶硅太阳能电池片及组件等30个重点项目，确保取得实质性进展。

大力发展服务业。

坚持把服务业作为推动产业结构优化升级、提升城市形象、改善人民生活的战略重点，拓宽领域，提升功能，创新业态，加速发展。优先发展生产性服务业，特别是加快发展现代物流业，突出抓好海港物流产业聚集区、北方钢铁物流一期等重点项目建设，积极谋划建立钢铁、煤炭、铁矿石等大宗商品电子交易平台，全面提升物流业发展规模和水平。加快发展生活性服务业，推动商贸流通等传统服务业转型升级，大力发展文化娱乐、体育健身、旅游休闲、社区服务等产业。以清东陵、唐山湾国际旅游岛等十大旅游项目建设为重点，推动旅游业大发展、快发展。拓宽服务业发展领域，培育壮大科技服务、信息咨询、总部经济、会展经济等高端服务业。继续推进工业企业主辅分离，加快服务环节外包、剥离和转型。落实国家房地产调控政策，促进房地产业健康发展。进一步完善落实鼓励支持服务业发展的政策措施，确保全年服务业增加值增长13%。

加快打造“行业巨人”和“航空母舰”。

加大对优势产业、重点企业扶持力度，落实国家鼓励实体经济发展的各项措施，推进企业做大做强和产业集群发展。实施大型企业集团培育工程，力争到“十二五”末，在工业领域打造开滦、唐钢、冀东3个年营业收入超千亿的特大型企业集团，渤海钢铁、高速动车等5个超500亿的大型企业，三友、冀东油田等10个超200亿的大型企业;在服务业领域打造开滦国际物流、庞大汽贸等5个年营业收入超百亿的大型企业，唐山港等20个超50亿的大型企业，联旺经贸等50个超10亿的大型企业。实施中小企业成长工程，引导扶持一批创新型、成长型、科技型中小企业做优做强，力争到“十二五”末，打造年营业收入超亿元、拥有鲜明行业特色、在国内同行业领先的“小巨人”企业550家。发挥传统产业优势，促进产业资本与金融资本融合。高度重视品牌的创造、培育和经营，年内力争新增中国驰名商标4个以上。

(三)着力推进以曹妃甸为龙头的沿海开发开放，打造区域经济增长极。

紧紧抓住河北沿海地区发展规划上升为国家战略这一重大机遇，全面落实省加快推进曹妃甸开发建设现场办公会议要求和部署，调整完善发展思路，整合要素资源，着力深化改革开放，着力加快产业聚集，着力加强薄弱环节，突出重点、打造亮点，抢抓机遇、攻坚克难，举全市之力推进曹妃甸开发建设，开创沿海开发开放新局面。

进一步完善规划体系。

完善曹妃甸新区总体规划和产业发展规划、港口岸线开发利用规划、海洋利用和保护规划等专项规划，增强规划的科学性、前瞻性、法制性和权威性，通过规划科学引领曹妃甸新区的开发开放。

狠抓基础设施配套建设。

加快曹妃甸港区LNG码头、煤炭码头续建和京唐港区集装箱泊位等港口项目建设，进一步完善港口功能，唐山港货物吞吐量力争达到3.5亿吨。活化资产，加大投入，加快曹妃甸水、电、路、气、热等基础设施配套，突出抓好重点产业项目配套设施建设，集中力量抓好曹妃甸工业区综合服务区建设，促进人流、物流、信息流尽快聚集，为重大产业项目落地创造良好完备的生活设施和条件。稳步推进唐山湾生态城建设，完善唐海县城、南堡主城区服务功能。启动唐曹铁路规划建设。

加速产业聚集步伐。

把产业聚集作为曹妃甸开发最核心、最紧迫的任务，把招商引资、项目建设作为工作重点，集中精力，落实责任，强力攻坚。瞄准世界500强、央企、知名民营企业，加大招商引资力度，引进一批战略投资者，加快建设一批重大产业项目。突出抓好马自达自动变速箱、首钢长白机械制造等重点项目建设，特别是全力推进中石化千万吨炼油、首钢京唐钢铁二期、华润曹妃甸2×100万千瓦发电机组等一批重大产业支撑项目前期工作，力争早日开工建设。抓好中日唐山曹妃甸生态工业园建设，确保一批项目落地开工。

加快体制机制创新。

按照省要求，本着精简、效能、统一的原则，理顺曹妃甸新区管理体制，建立权责统一、分工合理、决策科学、执行顺畅、监督有力的新体制新机制。全面落实省、市支持曹妃甸新区发展的财税、融资、土地、用海、环保、行政审批等方面的各项政策措施，为曹妃甸的大开发、大发展创造良好环境。抓好曹妃甸国家级经济技术开发区和综合保税区申报推进工作，力争年内获批运行。

(四)着力推进“三农”工作，加快新农村建设。

坚持把改善农村生产生活条件和增加农民收入作为新农村建设的首要任务，进一步加大强农支农惠农力度，促进农业增效、农民增收、农村繁荣。

努力增加农民收入。

加大财政支农力度，全面落实粮食直补、农资综合直补等政策，确保财政支农资金增幅高于财政经常性收入增幅。在挖掘农业内部增收潜力的同时，加快发展非农产业特别是与农业生产相衔接的加工业，扩大农民非农就业。积极发展劳务经济，加快农村富余劳动力转移，增加农民工资性收入。

大力发展现代农业。

按照“一产抓特色”的思路，在稳定粮食生产的同时，进一步优化农业结构，强化科技支撑，加快产业化步伐，提升农业现代化水平。重点实施食品加工园区建设、设施农业提升、品牌创建等十大工程，提高农产品市场竞争力，促进农业生产经营专业化、标准化、规模化、集约化、品牌化，努力形成特色，提高效益。全市市级以上农业龙头企业达到280家以上，新发展农民专业合作社150个，争创国家名牌农产品或驰名商标1个以上，省名牌农产品或省著名商标5个，农业产业化经营率达到66%。

加强农村基础设施建设。

落实土地出让金收益10%用于农田水利的政策要求，加快实施以农田节水灌溉、抗旱水源工程为重点的农田水利基础设施建设，新增节水灌溉面积30万亩，恢复改善除涝面积25万亩。加强农村道路和通讯网络建设，实施新一轮农村电网改造，推进农村环境综合整治，开展村镇污水处理和城乡一体垃圾处理试点工作。积极稳妥推进新民居建设，新启动建设省级农村新民居示范村210个，总数达到1000个。

深化农村综合配套改革。

继续抓好统筹城乡发展试点工作。进一步推进农村产权制度改革，抓好农村集体土地所有权、乡镇企业集体用地使用权、农村宅基地使用权等确权颁证工作，完善土地承包经营权流转市场，积极发展多种形式的适度规模经营。扩大农村土地股份合作、农村资金互助合作试点。稳妥推进农村集体经济股份制改造，每个县(市)区抓1至2个试点。深化城乡户籍制度综合配套改革，促进农民向城镇有序转移。

(五)着力推进科技创新，增强经济发展内生动力。

把科技进步和创新作为转方式、调结构的中心环节，实施企业创新主体培育、高新技术产业集聚发展、创新平台建设、传统优势产业提升、战略性新兴产业培育、农业和民生科技创新示范、政产学研合作、知识产权创新等八项工程，深入推进国家创新型城市建设。市财政安排科技专项资金1.84亿元，比上年增长15.2%。

健全完善科技创新机制。

制定出台支持企业创新和科研成果转化等政策措施，健全完善科技创新考核评价和激励机制、知识产权保护机制。加大财政对科技的投入力度，确保全市用于科技的一般预算资金投入高于财政经常性收入增长比例。鼓励发展创业投资企业，建立多元化、多渠道的科技投入机制。支持企业建立工程技术研发中心，使企业成为创新投入的主体、产学研合作的主体、科技成果转化应用的主体。

加强创新平台建设。

发挥高新技术产业开发区和中科院唐山研究与转化中心示范引领作用，搭建科技创新研发、合作、服务、转化平台。着力推进产业技术研究院、工程技术研究中心、“两化”深度融合重点企业技术中心和重点实验室建设，加快高速动车组产业基地等一批特色产业基地发展，推进各级生产力促进中心做大做强。力争有3至5家科技研发机构晋升至国家或省级行列。

强化科技成果转化应用和推广。

坚持以企业为主体，以引进消化吸收再创新和集成创新为重点，围绕改造提升传统产业、发展战略性新兴产业和现代农业的重大关键共性技术需求，依托高等院校、科研院所，加大共性技术和关键核心技术的研究、示范和推广力度，实施一批重大科技专项，推广应用一批具有自主知识产权、与我市主导产业紧密衔接的新技术、新工艺、新产品。年内转化实施重大专利技术150项，申请专利量增长15%以上，开发省级以上新产品100项以上。

大力培养引进创新型人才。

继续实施领军人才选拔培养工程，培养造就一批推动科技创新和产业发展的高端人才。立足全市经济社会发展需求，发挥我市高校资源作用，加快高技能人才和专业技术人才培养。着眼于满足关键核心技术研究应用、高新技术和先进适用技术推广，加大高层次人才和急需紧缺专业人才的引进力度。完善人才使用管理和激励机制，对做出突出贡献的优秀人才实施重奖，营造人尽其才、人才辈出的良好环境。

(六)着力推进节能减排，加强环境保护和生态建设。

坚持环境优先、生态立市，正确处理经济增长、结构调整和节能减排三者关系，推进经济增长与环境保护和生态建设协调发展，加快建设资源节约型、环境友好型社会。

强力抓好节能减排。

严格落实节能减排责任制，健全节能减排统计、监测、考核体系。突出抓好新老“双三十”单位和“双百”企业节能减排，在钢铁、水泥、电力、焦化等七个行业广泛开展“能效领跑者”企业创建活动，实施余热蒸汽发电等100项节能技改项目和烧结机脱硫等267项减排项目。按照国家产业政策和省统一部署，坚决淘汰钢铁、水泥、造纸等行业落后产能。完善经济政策和市场机制，运用价格、税收、金融、排放权交易等经济手段，增强企业节能减排的内生动力。强化行业准入管理，严禁新上高耗能、高污染项目和低水平重复建设，做到“两高”项目一个不上、钢铁产能一吨不增。

大力发展循环经济。

以产品上下游衔接和能源资源深度利用为重点，在钢铁、水泥、化工等重点行业积极推广清洁生产和循环利用技术，加强循环经济示范园区建设，组织开展循环型企业创建，谋划实施一批循环经济示范项目。全市循环经济示范园区达到15个以上，每个县(市)区循环经济示范企业达到5个以上。

加强生态环境综合治理。

综合治理城市大气和水流域污染。加大海洋环境保护力度，抓好直接入海河流排污企业治理。深入推进矿山生态修复治理，大力开展植树造林，加强自然保护区、水源地、森林、湿地的保护和管理。有效防治农村面源污染，推进农村生活垃圾集中清运和无害化处理。抓好重点污染源环境安全监管，严格环境监督执法，切实解决好影响群众切身利益的突出环境问题。科学有序开发矿产资源，严厉打击各种违法开采行为。

(七)着力推进城镇建设上水平，加快建设繁荣舒适的生态宜居城市。

把城镇建设作为转方式、调结构的综合载体，以承办2016世界园艺博览会为契机，坚持外延拓展与内涵提升并重、建设与管理并重，不断提高城市规划建设管理水平，增强城市产业聚集力、经济辐射力和综合竞争力。

完善城市规划。

认真落实国务院批复的《唐山市城市总体规划(2011—2020年)》，进一步完善城市规划体系。完成市中心城市地下空间规划设计、市政基础设施规划、风貌特色规划编制，以及环城水系、凤凰新城、南湖生态城商业服务配套项目专项规划编制，组织编制城市综合交通规划。启动2016世界园艺博览会建设规划编制工作。建立城市规划与管理数字化信息系统，推进城乡地理信息系统建设。

提升城市功能。

加强城市基础设施建设，完成南新道、友谊路拓宽等道路改造工程，完善城市次干道和支路网络，开工建设西郊热电厂异地扩建项目，加快北郊、南郊热电项目前期工作，实施西郊、丰润热电联网供热和新华道等9条燃气管线扩容改造工程。加强公交场站建设，加快公交车辆更新升级，建立城市快速公交体系。加快新火车站东广场及周边区域改造，启动新火车站西广场及周边区域规划建设。加快新青少年宫、新科技馆、新工人文化宫等项目建设，高标准建成一批文化、科教、体育、卫生、养老等公共服务和便民设施，提高城市综合承载能力。继续推进旧小区改善和既有建筑节能改造，全部完成震后危旧平房改造安置住房开工建设任务。加快城中村和旧城改造回迁房建设步伐，确保拆迁居民及时回迁。

繁荣城市经济。

把发展服务业作为城市建设的重要内容，以环城水系周边区域、南湖生态城、凤凰新城、空港城为重点，高标准规划建设一批商业金融、物流商贸、高新技术、文化创意等产业聚集区、特色街区和商贸综合体，实现城市建设与产业发展良性互动。环城水系公园广场全部建成开放，在水系沿线规划建设一批大体量的商业服务、文化产业项目，打造生态景观带和产业聚集带。提升南湖生态城软硬件水平，完善南湖生态城基础设施和配套功能，加快老唐山风情小镇、影视基地、访客中心、国家体育休闲示范基地等项目建设，着力打造高品质生态旅游度假区和5A级景区。加快推进凤凰新城金融街、城市综合体、总部基地等现代服务业项目建设，提高城市繁荣度和产业聚集能力。继续完善空港城路网等基础设施配套，为产业聚集创造条件。

加强城市管理。

坚持建管并重，完善城市管理体制，理顺市、区、街道、社区城市管理职责和权限。健全城市管理综合执法体制，建立规划、国土、城管、住建等部门协调联动机制。深入推进城区和县城数字网格化管理，加强城市应急体系建设，全面提升城市科学化、精细化管理水平。持续开展城市“绿、美、亮、净”综合整治，进一步提升城市环境和形象。

加快县城扩容和小城镇建设。

坚持大中小城市协调发展，在做大做强中心城区的同时，扶持迁安、遵化、滦县、乐亭、滦南尽快建成中等城市，促进玉田、迁西、唐海县城扩容升级。加快县城景观示范街、标志性建筑和城中村改造步伐。加大特色重点镇建设力度，促进产业向园区聚集、园区向城镇集中、人口向城镇转移。

(八)着力推进改革开放，为经济社会发展注入活力和动力。

改革开放是破解体制机制障碍和发展难题、实现又好又快发展的根本途径。我们要以更大的决心和力度，进一步深化改革、扩大开放。

加快改革攻坚步伐。

深化国有企业改革，推进优势企业跨行业、跨所有制兼并重组，支持优势企业上市发展，力争新增上市公司3家以上，每个县(市)区着手培育1至2家上市公司。深化行政管理体制改革，巩固市县政府和乡镇机构改革成果，做好工商、质监行政管理体制调整。稳步推进事业单位分类改革，按照国家、省统一部署，力争上半年完成清理规范。深化财政体制改革，培植壮大财源，强化治税管费，构建综合治税格局，健全公共财政体制，优化财政支出结构，落实财政预算绩效管理，加强政府债务管理，确保财政持续增长、平稳运行。深化金融体制改革，支持市商业银行做大做强，支持市区5家农村信用联社和符合条件的县级联社改建为农村商业银行，加快发展小额担保贷款和农户小额信用贷款等金融服务，积极发展证券、期货、保险、担保、融资租赁等多种金融业态。

全方位扩大开放。

切实把扩大开放摆到全局工作的突出位置，引导各级各单位各企业强化大开放意识，倾注更多精力抓开放、促合作，构建起面向所有投资者，包括一二三产业、涵盖经济文化社会各个领域的全方位大开放格局。创新招商方式，完善招商机制，突出招商重点，广泛开展市场化招商、驻点招商、委托招商、以商招商、小团组招商、产业链招商和网上招商，引进一批战略投资者。突出抓好100个重点招商合作项目和30个重点已签约外资项目的落地开工。精心办好第十五届唐山中国陶瓷博览会等年度展会。全年实际利用外资力争增长30%。抓住京津冀区域经济一体化进程加快的有利时机，进一步加强与京津和央企在产业、资金、技术等领域的对接合作，深化与长三角、珠三角的联系与合作。全年引进市外资金力争增长30%以上。优化出口产品结构，加强出口基地建设，深度拓展国际市场。全市进出口总额增长13%，其中出口增长13%。加快“走出去”步伐，支持有条件的企业开展境外投资、海外发展。

高度重视支持民营经济发展。

放手放胆发展民营经济，全面落实鼓励支持民营经济发展的各项政策措施，坚持“非禁即入”，引导民营资本参与农业、工业、服务业各个领域的经济建设和社会事业发展。尤其是鼓励民营企业就地发展、二次创业，支持民营资本投资发展服务业和战略性新兴产业。健全完善民营企业融资担保、人才培训、科技信息、创业辅导等服务平台，推进中小企业网站建设，积极帮助中小微企业解决信息、资金、技术、用地等实际问题。实施民营企业家素质提升工程，建设一支有抱负、懂经营、善管理、敢创新、讲诚信的民营企业家队伍。深入推动全民创业，激发各类市场主体的创业热情和发展活力。

进一步优化发展环境。

组织开展“优化发展环境年”活动，在全市形成人人代表唐山形象、合力优化宽松环境的浓厚氛围。营造优质高效的政务环境，进一步清理减少和调整行政审批事项，加强各级行政服务中心和电子政务建设，推行网上申报受理审批，提高办事效率，提升服务质量;营造公平正义的法治环境，加大对干扰、阻挠、破坏发展环境案件的查处力度，坚决制止“三乱”现象，切实保护各类市场主体合法权益;营造开放优越的市场环境，完善市场监管体系，大力规范和整顿市场经济秩序;营造诚实守信的人文环境，健全社会信用体系，真正让广大投资者放心、安心、舒心。

(九)着力推进各项社会事业，统筹经济社会协调发展。

在保持经济平稳较快发展的同时，进一步加大社会事业投入力度，推动各项社会事业全面进步。今年全市财政投入社会事业资金增长34%。

优先发展教育事业。

全市财政安排教育资金73.4亿元，其中市本级14.8亿元，分别增长34.2%和16.8%。实施学前教育三年行动计划，抓好城市新建住宅小区幼儿园配套建设，完成农村闲置小学改建幼儿园100所、农村小学附设幼儿园100所。推进义务教育均衡发展，完善义务教育经费保障机制，农村义务教育生均公用经费提高100元。加快推进中小学校舍安全工程，目前全市还有81万平方米改造任务，年内确保90%开工，2013年底全部完成267万平方米改造任务。大力发展职业教育，完善继续教育体系，广泛开展各类职业技能培训，努力实现人人拥有一技之能目标。提升高等教育水平，支持特色学科、重点学科建设，本科生均公用经费标准由7000元提高到12000元。积极谋划推进各类教育资源整合，改善办学条件，提高教育教学水平。鼓励发展民办教育。加强中小学教师继续教育，打造高素质教师队伍。加快构建校园安全体系，加强校车安全管理和校园饮食卫生安全。

推动文化大发展大繁荣。

市本级安排文化发展资金2.06亿元，同比增长2.8倍。以不断满足人民群众日益增长的精神文化需求为根本出发点，实施“文化立市”战略，进一步提升文化综合实力。加强社会主义核心价值体系建设，大力弘扬以爱国主义为核心的民族精神、以改革创新为核心的时代精神和不甘落后、敢为人先，奋发向上、大有作为的唐山新时代精神。巩固扩大全国文明城市创建成果，完善长效机制，深入开展群众性精神文明创建活动。扎实推进文化惠民，广泛开展多种形式的群众性文化活动，加快市文化广场、新工人文化宫等重点文化基础设施建设，加强县文化馆、图书馆，乡镇综合文化站，村“农家书屋”和社区文化中心、文化活动室建设，各级公共图书馆、群艺馆、文化馆等全部免费开放，丰富群众文化生活。办好第八届中国评剧艺术节、市第二届群众文化艺术节。大力发展文化产业，实施开放带动、项目拉动，引进和培育文化龙头企业，打造知名文化品牌。启动推进唐山交大遗址保护等20个重点文化产业项目建设，加快唐人文化园、启新记忆文化创意产业园、曹雪芹文化园等在建项目进度。以节庆活动、文艺创作、名人名家、地域文化为重点，打造独具唐山特色的系列文化品牌。深化文化体制改革，推动唐山演艺集团等转企改制单位建立现代企业制度，深化文化馆、图书馆等公益文化事业单位改革。推进重点媒体机制改革，进一步创新管理和运营机制。鼓励支持非公有制资本进入文化产业领域。

加快医疗卫生事业改革发展。

市本级安排医疗卫生事业发展资金9.7亿元，其中医改资金2.5亿元，同比分别增长13.2%和3.3倍。完善区域卫生发展规划。继续推进医药卫生体制改革，加快以县级医院为重点的公立医院综合改革，落实好基本药物制度，全面推进乡镇卫生院和村卫生室一体化管理。进一步提高新农合筹资标准和报销补偿比例，报销封顶线不低于8万元，参合率达到98%以上。实施“树名医、建名科、创名院”工程，不断提高医疗管理服务水平。继续开展“健康唐山，幸福人民”行动，为45岁以上农村居民和城镇无业居民免费体检，对筛查出的慢性病病人全部实施有效干预。加快市新工人医院、新妇幼医院建设，加强县乡村医疗机构建设。实施公共卫生保障工程，有效预防重大疾病和传染病，增强突发公共卫生事件应急处置能力。

全面发展体育、人口和计生等各项事业。

广泛开展全民健身运动，提高竞技体育水平，办好市第八届青少年运动会，加快市奥体中心建设。继续做好人口和计生工作，综合治理出生人口性别比问题，加强流动人口服务管理，稳定低生育水平。依法保障妇女和未成年人权益。认真落实老年人优待政策，加快社会养老机构建设，促进老龄事业发展。高度重视残疾人事业，推进残疾人社会保障、社会服务、法制制度三大体系建设，实施住房保障、生活保障、就业保障、康复治疗四项工程，促进残疾人事业全面发展。贯彻党的民族宗教政策，依法管理宗教事务，促进民族团结。加快广播电视、外事侨务、慈善、气象、防震减灾、档案、地方志等各项事业发展。

(十)着力推进民生改善，促进社会和谐稳定。

以人为本，民生为重。坚持把保障和改善民生作为一切工作的出发点和落脚点，以解决人民群众最关心、最直接、最现实的利益问题为着眼点，下大力保障和改善民生，让人民群众生活得更幸福、更有尊严。今年市级财政安排民生方面的支出58.7亿元，占一般预算支出比重达到75%。

千方百计扩大就业。

实施更加积极的就业政策，健全市场主导就业、政府促进就业、自谋职业、自主创业相结合的有效机制，多渠道开发就业岗位。落实鼓励高校毕业生就业各项扶持政策，积极开展就业推进行动，促进高校毕业生就业。深化退役士兵安置改革，做好退伍转业军人培训安置工作。完善就业援助机制，积极帮助就业困难人员实现稳定就业。加强创业资金、创业培训、孵化基地建设，促进以创业带动就业。建立健全与城乡社会经济发展需要相适应的职业培训体系，增强劳动者的就业能力。完善职工工资调整机制，推进工资集体协商和集体合同制度，构建和发展和谐稳定的劳动关系，依法维护劳动者合法权益。

完善社会保障体系。

以非公有制经济组织从业人员、个体劳动者、灵活就业人员和农民工为重点，推进社保扩面，实现城镇居民养老保险制度全覆盖和新型农村社会养老保险适龄人口全覆盖。抓好城镇基本医疗保险市级统筹工作，做好工伤保险省级统筹准备。继续开展职工重大疾病医疗互助活动。稳步提高社会保障标准，继续提高企业退休人员基本养老金。完善城乡低保标准自然增长机制，在动态管理、应保尽保的前提下，保障城乡困难群众生活水平同步提高。切实做好稳控物价工作，健全社会救助和保障标准与物价上涨挂钩联动机制。

继续为群众办一批实事。

今年，集中力量办好10件实事：一是努力扩大就业规模。新增城镇就业9万人以上，向非农领域转移农村富余劳动力12万人，“零就业”家庭动态归零，征集推荐1万个高校毕业生就业见习岗位。二是加快保障性住房建设。市中心区新开工建设保障性住房25万平方米，着力解决中低收入家庭住房问题。三是继续实施农村饮水安全工程。以保障水源安全和自来水入户为重点，解决好134个村、12.05万人的饮水安全问题。四是进一步提高城乡低保标准，城镇由每人每月363元提高到410元，农村由每人每年1900元提高到2300元。五是建立新农合大病统筹基金，最高支付限额30万元，有效缓解农民大病治疗难问题。六是解决市区群众乘车难问题。在市中心区投放出租车700辆至1000辆，新增公交车100部，同时进一步加强城市交通秩序管理。七是实施城乡社会养老幸福工程，建设1766个居家养老服务中心(站)。八是加大农村残疾人危房改造力度，对全市农村贫困残疾人危房户、无房户全部进行改建。九是完善提升社区惠民便民服务功能。规范建设20个和谐示范社区，改造提升100个城市社区;加强社区服务信息平台建设，满足社区居民家政、养老、保洁、医疗等多种服务需求;建设社区科技防范体系，为社区居民提供安全环境。十是稳步推进市中心区已完成改造的城中村居民参加城镇职工基本养老保险，优先保障已达到或超过退休年龄人员享受基本养老保险待遇。

维护社会和谐稳定是人民群众安居乐业的基础，是各级政府义不容辞的重大责任。创新社会管理，深入实施社会矛盾综合调控，更加注重社会矛盾源头预防治理，认真做好群众来信来访工作，发挥好市民公共服务热线作用，切实解决关系群众利益的突出问题。推进依法治市，扎实开展“六五”普法。加强社会治安综合治理，完善社会治安科技防范体系，继续深入开展“平安唐山”创建活动，尤其是加强对校园周边、公共复杂场所等重点区域部位的治安整治，依法严厉打击黑恶势力和各类违法犯罪活动，努力提高人民群众的安全感。毫不放松地抓好安全生产，全面落实安全生产责任制和各类安全防范措施，持续不断地开展重点领域和高危行业安全生产专项整治，坚决防止重特大事故发生，确保安全生产形势持续稳定。充分发挥市县两级政府食品安全委员会及其办公室职能作用，将所有食品全部纳入监管范围，深入开展食品药品安全专项整治，强化食品药品安全长效机制建设，让人民群众吃得放心、用得安全。

各位代表，做好今年经济社会发展各项工作，任务繁重、意义重大。我们将统筹兼顾、突出重点，严明责任、强化措施，把各项任务一项一项落到实处，务求取得实实在在的成效。圆满完成今年的工作任务，我们就会朝着科学发展、争先进位的目标迈出坚实一步，为走在全省改革发展前面打下坚实基础!

四、切实加强政府自身建设

适应新形势、新任务要求，全面完成今年经济社会发展各项目标任务，必须以转变政府职能、提高行政效能为核心，进一步加强政府自身建设。

强化责任意识，解放思想，加压奋进。面对当前复杂多变的新形势，面对走在全省改革发展前面的新要求，面对广大人民的新期盼，认清肩负的重大责任和使命，始终保持奋发向上、大有作为的精神状态，敢于担当、勇挑重担，负重奋进、奋勇争先。深入开展以“如何走在全省改革发展前面”为主题的解放思想大讨论，进一步推进思想大解放。特别是紧密结合唐山改革发展实际，围绕解决突出矛盾、破解发展难题，创新理念、创新方法、创新举措，不断提高推动科学发展、服务人民群众的本领和能力。

强化实干意识，求真务实，埋头苦干。

大力发扬求实精神和务实作风，出实招、用实劲，求实效、重实绩，在全市各级政府机关努力营造崇尚实干、埋头苦干的浓厚氛围。心无旁骛抓落实，集中精力抓重点、攻难点、搞突破，把各项工作扎扎实实推向前进。进一步健全完善认真负责、层层负责、科学合理、行之有效地抓落实工作机制，全面落实目标管理责任制，推行政府绩效管理和行政问责制，形成干事创业的合力。

强化服务意识，改进作风，一心为民。

始终坚持把服务群众作为政府的首要职责，把人民群众的利益放在首要位置，真正做到权为民所用，情为民所系，利为民所谋。加快政府职能转变，完善公共服务体系，将公共资源更多地向民生领域倾斜，更好地为基层、为企业、为群众服务。增强服务意识和效率观念，进一步精简审批事项，规范审批程序，减少审批环节，下大力解决行政不作为、乱作为问题，努力提升行政效能和服务水平，营造良好发展环境。坚持深入基层、深入实际，注意倾听群众呼声，真心实意地为人民群众办实事、解难事。

强化法制意识，公开公正，依法行政。

自觉接受人大法律监督、政协民主监督和社会监督，完善和加强政府内部监督，确保行政权力依法公正行使。深入推进行政权力公开透明运行，自觉接受群众监督。建设行政审批电子政务系统和行政权力运行电子监察系统。健全社情民意反映制度和重大事项专家咨询、社会公示、听证质询制度及行政决策风险评估机制，切实做到科学决策、民主决策、依法决策。严格按照法定权限和程序行使职权、履行职责，自觉运用法律手段调节经济和社会事务。加强行政立法，强化行政执法监督，严格落实行政执法责任制，坚决防止违法施政现象发生。

强化廉政意识，从严治政，廉洁从政。

认真落实党风廉政建设责任制，深入推进政府系统惩治和预防腐败体系建设。坚持不懈开展党风廉政教育，教育引导政府系统广大干部堂堂正正做人，干干净净做事。进一步健全完善行政权力运行监控机制，切实加强对重点项目建设、国有资产经营管理、建筑工程招投标、行政审批和直接关系人民群众切身利益、人民群众高度关注的领域和行业的监督管理，从源头上预防和减少腐败现象发生。加大查办违纪违法案件力度，严厉惩治各种腐败行为。大力倡导艰苦奋斗、勤俭节约，严格控制“三公”经费支出，坚决反对铺张浪费和奢靡之风，塑造政府良好形象。

各位代表，回顾2011年我们取得了可喜成绩，展望2012年我们满怀信心。让我们紧密团结在以胡锦涛同志为总书记的党中央周围，在省委、省政府和市委的正确领导下，同心同德，真抓实干，全力推动唐山科学发展、争先进位，努力走在全省改革发展前面，以优异成绩迎接党的十八大胜利召开!
